# A Rare Case of Mosaic 3pter and 5pter Deletion-Duplication with Autism Spectrum Disorder and Dyskinesia

**DOI:** 10.1155/2023/7974886

**Published:** 2023-10-16

**Authors:** Luna Bajracharya, Meena Lall, Sunita Bijarnia-Mahay, Praveen Kumar, Imran Mushtaq, Pushpa Saviour, Preeti Paliwal, Anju Joshi, Shruti Agarwal, Praveen Suman

**Affiliations:** ^1^Department of Pediatrics, Maharajgunj Medical Campus, Institute of Medicine, Tribhuvan University Teaching Hospital, Kathmandu, Nepal; ^2^Institute of Medical Genetics and Genomics, Sir Ganga Ram Hospital, New Delhi, India; ^3^Department of Pediatric Neurology, Institute of Child Health, Sir Ganga Ram Hospital, New Delhi, India; ^4^Child Developmental Clinic, Institute of Child Health, Sir Ganga Ram Hospital, New Delhi, India

## Abstract

**Introduction:**

There is evidence that neurodevelopmental disorders are associated with chromosomal abnormalities. Current genetic testing can clinch an exact diagnosis in 20–25% of such cases. *Case Description.* A 3 years and 11 months old boy with global developmental delay had repetitive behaviors and hyperkinetic movements. He was stunted and underweight. He had ataxia, limb dyskinesia, triangular face, microcephaly, upward slanting palpebral fissure, hypertelorism, retrognathia, posteriorly rotated ears, long philtrum, thin lips, broad nasal tip, polydactyly, tappering fingers, and decreased tone in the upper and lower limbs with normal deep tendon reflexes. Magnetic resonance imaging of the brain, ultrasound of the abdomen, and ophthalmological evaluation were normal. Brain evoked response auditory revealed bilateral moderate hearing loss. He fulfilled the Diagnostic Statistical Manual 5 criteria for autism. In the Vineland Social Maturity Scale, his score indicated a severe delay in social functioning. His genetic evaluation included karyotyping, fluorescence in situ hybridization (FISH), and chromosomal microarray analysis (CMA). The karyotype report from high-resolution lymphocyte cultures was mos 46, XY, der(3)t(3; 5)(p26; p15.3)[50]/46, XY,der(5) t(3;5) (p26;p15.3)[50].ish. His karyotype report showed a very rare and abnormal mosaic pattern with two cell lines (50% each). Cell-line#1: 3pter deletion with 5pter duplication (3pter−/5pter+) and cell-line#2: 3pter duplication with 5pter deletion (3pter+/5pter−) derived from a *de novo* reciprocal translocation t(3; 5)(p26; p15.3) which was confirmed by FISH. The chromosomal microarray analysis report was normal. The two cell lines (50% each) seem to have balanced out at the whole genome level. Occupational, sensory integration, and behavior modification therapy were initiated for his autistic features, and anticholinergic trihexiphenidyl was prescribed for hyperkinetic movements.

**Conclusion:**

This case highlights a rare genetic finding and the need for timely genetic testing in a child with dysmorphism and autism with movement disorder to enable appropriate management and genetic counselling.

## 1. Introduction

Genetic causes have been identified as an underlying factor in many neurodevelopmental disorders [1]. Global developmental delay, autism, behavioral problems, speech impairment, seizures, etc., are implicated in syndromes associated with chromosomal abnormalities [2].

Here, we present a rare case of a boy with dysmorphism, autism spectrum disorder (ASD) and dyskinesia who harbored a unique mosaic deletion-duplication pattern involving the terminal parts of chromosomes 3p and 5p. A combined classical approach of karyotyping, FISH, and chromosome microarray analysis was used to resolve this rare chromosomal abnormality in order to render personalized genetic counselling to the parents for their future pregnancies.

## 2. Case Presentation

**Figure 1:**
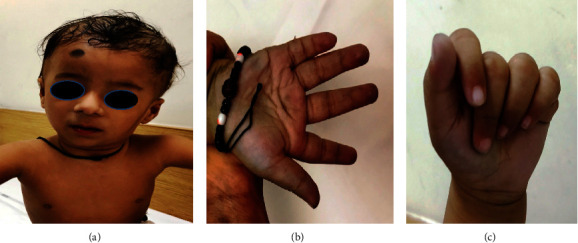
(a) Facial dysmorphism (triangular face, hypertelorism, broad nasal tip, long philtrum, thin lips, retrognathia, and posteriorly rotated ears) in proband at 10 months. (b) Tapering fingers (right hand). (c) Tapering fingers (left hand). Small bump near the lateral region of the left little finger was the spot where a small postaxial finger was excised.

The boy is the second born of a nonconsanguineous marriage. He was born via normal vaginal delivery at term, weighing 2500 grams, without any significant perinatal history. He presented with delayed developmental milestones and dysmorphism in a superspeciality tertiary care hospital, India. At 10 months, he could sit momentarily with support, bring his hand in midline to hold objects, and babble. There was no history of fever, rash, vomiting, loss of consciousness, or head trauma. There was no family history of developmental delay, seizure, or congenital abnormalities. On examination at 10 months of age, he was noted to have occipitofrontal circumference of 42.5 cm (*z* score: −2.69), weight of 6.5 kg (*z* score: −3.86), and length of 73 cm (*z* score: −0.24). His facial features included triangular face, hypertelorism, broad nasal tip, long philtrum, thin lips, retrognathia, and posteriorly rotated ears (Figure 1(a)), the right postaxial tiny rudimentary polydactyly and tapering fingers (Figures 1(b) and 1(c)). The child had an excision of a small postaxial left finger at the age of 8 months (Figure 1(c)). There was hypotonia with normal deep tendon reflexes and no abnormal body movements. No organomegaly or any neurocutaneous stigmata were noted. The external genitalia and spine were normal.

**Figure 2:**
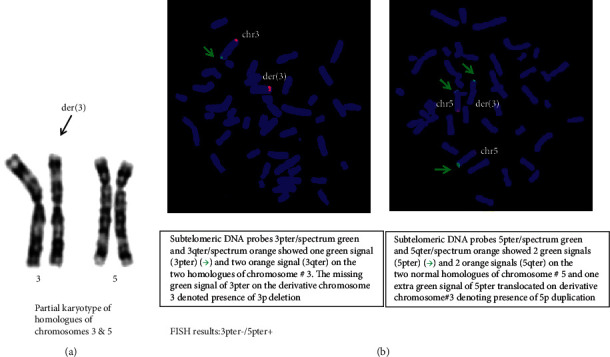
Cell-line #1: karyotype: 46, XY, der (3) t (3; 5) (p26; p15.3).ish. (a) Partial karyotype of chromosomes 3 and 5 showing derivative der (3) t (3, 5) (p26; p15.3). (b) FISH analysis which showed 3pter−/5pter+.

**Figure 3:**
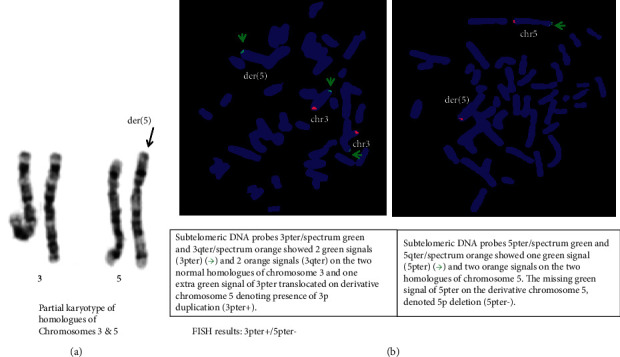
Cell-line #2: karyotype: 46, XY, der (5) t (3; 5) (p26; p15.3).ish. (a) Partial karyotype of chromosomes 3 and 5 showing derivative der (5) t (3, 5) (p26; p15.3). (b) FISH analysis which showed 3pter+/5pter−.

Genetic evaluation in the child included a karyotype, fluorescence in situ hybridization (FISH), and chromosomal microarray analysis (CMA) [3–5]. The karyotype report was mos 46, XY, der (3) t (3; 5) (p26; p15.3)[50]/46, XY, der (5) t (3;5) (p26;p15.3)[50].ish. The high resolution lymphocyte cultures of the peripheral blood sample of the proband had two cell lines causing mosaicism. Out of hundred metaphases examined, fifty (50%) had cell-line #1 which showed presence of a derivative chromosome der (3) t (3; 5) (p26; p15.3) causing deletion 3pter and duplication 5pter (3pter-/5pter+) which was confirmed by FISH (Figures 2(a) and 2(b)), and the remaining fifty metaphases (50%) consisted of cell-line #2, which showed presence of the derivative chromosome der (5) t (3; 5) (p26; p15.3) with exactly opposite rearrangement of duplication 3pter and deletion 5pter (3pter+/5pter−) which was also confirmed by FISH (Figures 3(a) and 3(b)). Subtelomeric DNA probes for chromosomes 3 and 5 used for FISH consisted of 3pter/spectrum green, 3qter/spectrum orange, 5pter spectrum green, and 5qter/spectrum orange. This chromosomal constitution was denovo as the parents peripheral blood had normal karyotypes and normal FISH results.

After 4 months when the child revisited the genetic clinic for a follow up, repeat karyotyping on the blood sample was done which reconfirmed this rare finding. No other tissues were studied as the parent did not consent.

**Figure 4:**
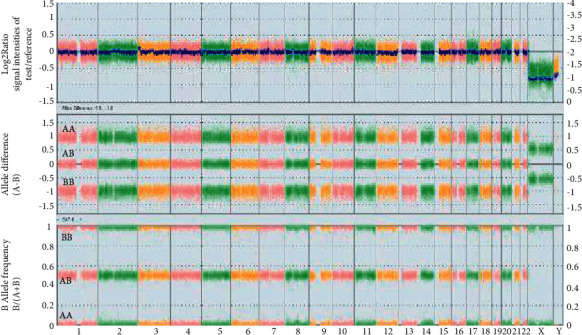
Whole genome view: Log2Ratio, allele difference, and B allele frequency displayed normal pattern from one genome.

The Affymetrix 750K microarray platform with ChAS software was used to calculate Log2Ratio which is the ratio of signal intensities between the test sample and the reference to detect copy number deletions and duplications [5]. Interestingly, CMA result of the proband was normal, not revealing any copy number variations specifically related to chromosomes 3 or 5. The whole genome view of Log2Ratio, allele difference (A−B), and B allele frequency (B/(A + B)) displayed normal pattern from one genome (Figure 4). This also ruled out the option of chimerism. A mosaic contains genetically different cells, originating from a single zygote, and chimerism occurs if there are genomes from two zygotes in one individual. SNP array helps to differentiate between mosaicism and chimerism, even in the absence of sex chromosome differences in the two coexisting cell lines [6]).

At 15 months of age, brain evoked response auditory (BERA) diagnosed bilateral hearing loss, for which he was using hearing aids intermittently. His visual assessment was normal. The child could not come for regular hospital visit due to COVID-19 pandemic and he presented again at 3 years and 11 months at the Child Development Clinic at our hospital with repetitive behaviors such as stacking or lining up the toys and abnormal limb movements along with developmental delay. There was no loss of consciousness, frothing from mouth, tongue bite, and urinary or stool incontinence during those episodes. There was no history of aggravating factors such as looking at bright toys, or loud sound associated with these movements. They did not occur during sleep. There was no history of head injury, icterus, rash, drug intake, or toxin exposure before onset of these abnormal movements. There were no similar complaints in any family members.

On examination, the child was aloof with facial dysmorphism, as described earlier. He walked with a broad-based gait. Both axial and appendicular ataxia was observed. At times, repetitive rocking movements of trunk and bilateral upper and lower limbs dyskinesias were evident. He displayed nonrhythmic, rapid, stereotyped hand movements as well. Clinically, it appeared to be nonepileptic. His weight was 14 kg (*z* score −1.07), height of 96.5 cm (*z* score −1.30), and occipital frontal circumference as 45.7 cm (*z* score −3.03). He did not respond to his name and had poor eye contact. He looked from corner of eyes and produced humming sound. In gross motor skills, he could walk up and down stairs with support, scribbled in imitation, and cooperated with dressing. He did not play with other children in waiting area in the clinic. He neither had protodeclarative nor protoimperative pointing. He started saying bisyllables as “mama” at 18 months of age but could not say any other meaningful words. Cranial nerves, bulk, and deep tendon reflexes were within normal limits except a decrease tone in the upper and lower limbs. No other cerebellar signs such as nystagmus were noted. Other systemic examinations were normal. His total developmental age and developmental quotient was 13.93 months and 29.63, respectively, in the Gesell developmental schedule. He fulfilled the Diagnostic Statistical Manual 5 (DSM-5) criteria for autism spectrum disorder (ASD). His score on Indian Scale for Assessment of Autism (ISAA) fell into moderate autism category. In Vineland Social Maturity Scale (VSMS), his social age was 14.1 months, which corresponds to the social quotient of 30 that indicated severe delay in social functioning of the child.

His electroencephalograph (EEG) showed no spikes or sharp waves. Magnetic resonance imaging (MRI) of brain, abdominal ultrasound, echocardiography, and ophthalmological evaluation were normal. His biomedical analysis of the blood and urine showed no obvious abnormality.

He was advised occupational, sensory integration, and behavior modification therapy for his autistic features. He was receiving two antiseizure medications, sodium valporate, and levetiracetam. Since abnormal limb movements were found to be clinically nonepileptic and with unremarkable EEG, his antiseizure medications were gradually weaned off. Trihexiphenidyl was prescribed for limb dyskinesias. During a phone call after four weeks of therapy, his parents informed that he had a better eye contact with reduced frequency and duration of repetitive hand movements. Parents were advised for repeat EEG and further evaluations if semiology of these movements change.

## 3. Discussion

The proband was diagnosed with a unique mosaic combination of chromosome 3pter and 5pter deletion and duplication, with overlapping clinical features consistent with 3pter−, 3pter+, 5pter−, and 5pter+ syndrome, as shown Table 1. A summary of clinical synopsis of chromosome 3pter deletion (OMIM: 613792), 3pter duplication (ORPHA: 96071), 5pter deletion (OMIM: 123450), and 5pter duplication (ORPH: 1742) syndromes is available in Online Mendelian Inheritance in Man (OMIM) and Orphanet Rare Disease Ontology [7–10]. The table also shows that the proband had overlapping features such as a triangular face, microcephaly, down turned lips, micrognathia, long philtrum, low set deformed ears, broad nasal tip, and polydactyly. The four chromosomal abnormalities (3pter−, 5pter+, 3pter+, and 5pter−) may also be associated with congenital heart disease and gastrointestinal and renal manifestations which was not evident in the proband, who may have a milder manifestation of the four syndromes due to presence of mosaicism. Sensorineural hearing loss can also occur in chromosome 3pter and 5pter deletion, as described in Table 1. The phenotype of the child closely resembles 3p deletion rather than 3p duplication, 5p deletion, or 5p duplication.

In this case, mosaicism of cell lines with deletion and duplication of 3pter and 5pter were identified at 10 months of age, which is very rare and adds to the existing literature. The deletion-duplication syndrome of chromosome 3 has been described in two fetuses and detected on prenatal chromosomal analysis via amniocentesis. Fetal testing was done for fetal lumbosacral meningocele in one case and for advanced maternal age in the other [11, 12]. In a later case, pregnancy was terminated at 24 weeks and the fetus was noted to have a triangular face, hypertelorism, a depressed nasal bridge, anteverted nostrils, long philtrum, downturned mouth, low-set ears, and clinodactyly of the hands [12]. Another case was a 17 -month-old girl with a supernumerary nipple, ptosis, microcephaly, epicanthal folds, broad nasal bridge, smooth philtrum, heart defect, umbilical hernia, urachal remnant, gross motor delay, and hypotonia. Her microarray analysis revealed a 5.37 Mb deletion of chromosome bands 3p26.1 to 3p26.3 and a 13.68 Mb duplication of 3p24.3 to 3p26.1. FISH analysis confirmed that the duplication was inverted in all the cells with no mosaicism [13]. A terminal deletion and inverted duplication of chromosome 5p have been described in 7 cases so far [14]. The phenotypic findings in all these reports are associated with the size and gene content of their deletions and duplications. To date, there are no reports of postnatal diagnosis of mosaic deletion-duplication combination of chromosomes 3p26 and 5p15, as reported presently.

Though chromosomal analysis revealed the 3pter, 5pter deletion, and duplication syndrome before 1 year of age, associated neurodevelopmental disorders such as autism spectrum disorder, developmental delay, speech delay, seizure, and ataxia manifested later in our case, similar to those reported in the literature [15–19].

Terminal deletion and duplication at 3p26.3 including *CHL1* (Cell Adhersion Molecule Like 1) are associated with language and cognitive delay. *CHL1* is highly expressed in the central and peripheral nervous systems [20].

Deletions in *ITPR1* (inositol-triphosphate receptor type 1) on chromosome 3p26.1 are associated with spinocerebellar ataxia [21]. The child had ataxic gait and hypotonia without brisk deep tendon reflexes. The deletion 3pter in the proband may have contained ITPR1 gene in our case, which can explain the reason for ataxia. Onset of this disorder can be early, sometimes slow, or nonprogressive [21, 22]. Our patient displayed ataxia after 3 years of age.

Deletions of variable size of 5pter cause Cri du chat syndrome; the clinical manifestation is variable, depending on the size of the deletion and gene content [9]. A report of atypical Cri du chat syndrome showing a 5p15.3 deletion suggests that the characteristic cry of the cat in their case was attributed to this distal 5p15.3 deletion [23]. However, our proband did not manifest any such cry at birth. This may be due to the mosaic status of his chromosome constitution.

The proband also had some overlapping features with 3pter+ and 5pter+ (Table 1). Phenotypes manifested by duplications may be milder than those caused by loss-of-function due to haploinsufficiency caused by deletion, and so are often missed or underreported [24].

Clinically, at times, like in our case, nonepileptic events may be mistaken for a seizure, so a caution is warranted. Comprehensive early interventions such as occupational, sensory integration, and behavioral modification therapies can improve the quality of life of children with autism associated with chromosome abnormalities [25, 26]. Parents of the child opted for these age-appropriate individualized early interventions and stimulations that improved the overall development of the child, including social interactions. Due to the clinical complexity of autism, the final diagnosis is often delayed. Detailed genetic evaluation can enhance an early, correct, and precise diagnosis for timely management.

Presently, we report a proband with two abnormal cell lines cultured from the blood sample. To the best of our knowledge, this is the first report presenting mosaicism of two cell lines: one cell line (50%) with derivative chromosome der (3) t (3; 5) (p26; p15.3) causing 3pter−/5pter+ and the second line (50%) with der (5) t (3; 5) (p26; p15.3) causing 5pter−/3pter+. The two cell lines seem to have derived from a de Novo balanced translocation t (3; 5) (p26; p15.3) which was confirmed by FISH. This case brings out the limitations of CMA in that it does not detect complementing rearrangements (i.e., deletion as well as duplication of the same chromosomal segment) occurring in mosaic pattern, especially in similar proportion (50% each), and reports it as normal, as in the interpretation of CMA with balanced translocations [27]. This worked to our advantage to infer that the overall genome report in the blood sample was balanced off by CMA, although the abnormal cell lines have created a pathological clinical outcome in the proband.

The exact molecular mechanism of this rare genetic finding is unknown. Presence of *de Novo* structural chromosomal abnormalities resonates that one parent may be a carrier of gonadal balanced translocation [28]. Therefore, the rare *de novo* mosaicism may have resulted from meiotic error during gametogenesis followed by mitotic error during the first blastomeric division at the early stage of embryogenesis [3, 29]. The parents were counselled for potential reproductive recurrence risk due to possible gonadal mosaicism, management of subsequent pregnancies and prenatal diagnosis. An approach of combining complementary classical genetic tests has been used even earlier to resolve rare cytogenetic findings [30]. The geneticist would discuss options for a noninvasive NIFTY-pro test which enables the identification of 6 autosomal aneuploidies (chromosomes 13, 18, 21, 9, 16, and 22), sex chromosomes aneuploidies, and 84 microdeletion syndromes [31], or invasive tests of amniotic fluid or chorionic villus for culture and subsequent karyotyping, combined with FISH and microarray analysis to render reproductive counselling for future pregnancies of the parents.

## 4. Conclusion

We report a very rare mosaic of 3pter and 5pter deletion-duplication. Apart from dysmorphism and global developmental delay, the child also presented with sensorineural hearing loss, autism spectrum disorder, ataxia, and limb dyskinesias. The child displayed characteristic clinical features of 3pter and 5pter deletion and duplication which have not been reported till date. The genetic rarity of this case was revealed using a combination of complementary genetic methods. Thus, this case highlights the need for timely genetic evaluation using a combination of genetic tests and appropriate early intervention to improve the quality of life of the child, and also counselling of parents for the management of the patient, and future reproductive risks and options.

## Figures and Tables

**Table 1:** Comparison of clinical features of patient with chromosome 3p deletion, 3p duplication, 5p deletion, and 5p duplication [7–10].

	3p deletion OMIM: 613792	3p duplication ORPHA: 96071	5p deletion OMIM: 123450	5p duplication ORPHA: 1742	Proband
Growth					
Low birth weight	+		+		−
Short stature	+		+		+
Head					
Microcephaly	+	+	+		+
Brachycephaly	+	+	+		−
Trigonocephaly	+				−
Dolichocephaly				+	−
Macrocephaly				+	
Flat occiput	+				−
Face					
Shape of face	Triangular	Square	Round	Long	Triangular
Frontal bossing		+			−
Broad forehead		+			−
Micrognathia	+	+	+	+	−
Retrognathia	+	+			+
Phitrum	Long	Prominent		Long	Long
Mouth					
Downturned corners of mouth	+	+	+		+
Thin lips	+		+		+
High arched palate	+		+		−
Dental malocclusion			+		−
Ears					
Low-set/dysmorphic	+	+	+	+	+
Hearing loss	+		+		+
Eyes					
Palpebral fissures	Upslanting		Downslanting		Upslanting
Hypertelorism	+	+	+		+
Epicanthal folds	+		+	+	−
Strabismus	+		+		−
Synophrys	+				−
Ptosis	+				−
Myopia			+		−
Nose					
Broad nasal bridge	+	+	+		−
Broad nasal tip	+				+
Heart					
Atrioventricular defect	+		+		−
Patent ductus arteriosus		+	+		−
Abdomen					
Feeding problems	+		+		−
Genitalia	Cryptorchidism in males	Hypogenitalism			Normal
Renal malformation	+	+			−
Skeletal					
Cranial sutures	Prominent metopic suture				Normal
Postaxial polydactyly	+				+
Fingers	Tapering		Short fifth finger clinodactyly	Long	Tapering
Sacral dimple	+				−
Spine			Scoliosis/kyphosis		−
Muscle, soft tissue					
Hypotonia	+		+	+	+
Spasticity	+		+		−
Neurologic/behavioral-psychiatric					
Ataxic-like broad-based gait			+		+
Psychomotor retardation	+	+		+	+
Delayed speech	+				+
Seizure	+	+		+	−
Attention deficit hyperactivity disorder			+	+	−
Autism spectrum disorder			+		+
Sleep disturbances			+	+	−
Voice					
High pitched cat‐like cry			+		−
MRI head findings			Hypoplastic/agenesis of the corpus callosum, periventricular leukomalacia, hydrocephaly, cerebral/cerebellar atrophy	Hydrocephalus/agenesis of the corpus callosum/Dandy–Walker malformation	−

+ indicates presence of clinical features. −indicates absence of clinical features. Blanks indicate features not reported.

## Data Availability

The data used to support the findings of this study are available from the corresponding author upon request.

## References

[1] Retterer K., Juusola J., Cho M. T. (2016). Clinical application of whole-exome sequencing across clinical indications. *Genetics in Medicine*.

[2] Hu T., Zhang Z., Wang J. (2019). Chromosomal aberrations in pediatric patients with developmental delay/intellectual disability: a single-center clinical investigation. *BioMed Research International*.

[3] Lawce H. J., Sanford J., Arsham M. S., Barch M. J., Lawce H. J. (2017). Cytogenetics: an overview. *The AGT Cytogenetics Laboratory Manual*.

[4] Lawce H. J., Sanford J., Arsham M. S., Barch M. J., Lawce H. J. (2017). Fluorescence in situ hybridization (FISH). *The AGT Cytogenetics Laboratory Manual*.

[5] Thermo Fisher (2023). Microarray analysis. https://www.affymetrix.com.

[6] Conlin L. K., Thiel B. D., Bonnemann C. G. (2010). Mechanisms of mosaicism, chimerism and uniparental disomy identified by single nucleotide polymorphism array analysis. *Human Molecular Genetics*.

[7] NCBI (2022). Chromosome 3pter-p25 deletion syndrome (#613792). Online mendelian inheritance in man (OMIM) database. http://www.ncbi.nlm.nih.gov/omim.

[8] ORPHA (2022). Distal trisomy 3p (ORPHA code: 96071). Orphanet rare disease Ontology. https://www.orpha.net/consor/cgi-bin/index.php.

[9] NCBI (2022). Chromosome 5p deletion syndrome (#123450). Online mendelian inheritance in man (OMIM) database. http://www.ncbi.nlm.nih.gov/omim.

[10] ORPHA (2022). Trisomy 5p (ORPHA code: 1742). Orphanet rare disease Ontology. https://www.orpha.net/consor/cgi-bin/index.php.

[11] Prabhakara K., Bruno D. L., Padman P. (2008). Prenatal detection of deletion-duplication of chromosome 3 arising from meiotic recombination of a familial pericentric inversion. *Prenatal Diagnosis*.

[12] Chen C. P., Su Y. N., Hsu C. Y. (2011). Mosaic deletion-duplication syndrome of chromosome 3: prenatal molecular cytogenetic diagnosis using cultured and uncultured amniocytes and association with fetoplacental discrepancy. *Taiwanese Journal of Obstetrics & Gynecology*.

[13] Riley J. D., Stefaniuk C. M., Erenberg F., Erwin A. L., Palange L., Astbury C. (2019). Chromosome 3p inverted duplication with terminal deletion: second postnatal case report with additional clinical features. *Case reports in genetics*.

[14] Krgovic D., Blatnik A., Burmas A., Zagorac A., Kokalj Vokac N. (2014). A coalescence of two syndromes in a girl with terminal deletion and inverted duplication of chromosome 5. *BMC Medical Genetics*.

[15] Cuoco C., Ronchetto P., Gimelli S. (2011). Microarray based analysis of an inherited terminal 3p26.3 deletion, containing only the CHL1 gene, from a normal father to his two affected children. *Orphanet Journal of Rare Diseases*.

[16] Kashevarova A. A., Nazarenko L. P., Schultz-Pedersen S. (2014). Single gene microdeletions and microduplication of 3p26.3 in three unrelated families: CNTN6 as a new candidate gene for intellectual disability. *Molecular Cytogenetics*.

[17] Tsuboyama M., Iqbal M. A. (2021). CHL1 deletion is associated with cognitive and language disabilities-case report and review of literature. *Molecular Genetics & Genomic Medicine*.

[18] Gandawijaya J., Bamford R. A., Burbach J. P. H., Oguro-Ando A. (2020). Cell adhesion molecules involved in neurodevelopmental pathways implicated in 3p-deletion syndrome and autism spectrum disorder. *Frontiers in Cellular Neuroscience*.

[19] De Silva D., Williamson K. A., Dayasiri K. C. (2018). Gillespie syndrome in a South Asian child: a case report with confirmation of a heterozygous mutation of the ITPR1 gene and review of the clinical and molecular features. *BMC Pediatrics*.

[20] NCBI (2022). Cell adhesion molecule L1-like; CHL1 # 607416 online mendelian inheritance in man (OMIM) database. http://www.ncbi.nlm.nih.gov/omim.

[21] NCBI (2022). Inositol 1, 4, 5-triphosphate receptor, type 1; ITPR1 (#147265) online mendelian inheritance in man (OMIM) database. http://www.ncbi.nlm.nih.gov/omim.

[22] Wang L., Hao Y., Yu P. (2018). Identification of a splicing mutation in ITPR1 via WES in a Chinese early-onset spinocerebellar ataxia family. *The Cerebellum*.

[23] Elmakky A., Carli D., Lugli L. (2014). A three-generation family with terminal microdeletion involving 5p15.33-32 due to a whole-arm 5; 15 chromosomal translocation with a steady phenotype of atypical cri du chat syndrome. *European Journal of Medical Genetics*.

[24] Yan J., Zhang F., Brundage E. (2009). Genomic duplication resulting in increased copy number of genes encoding the sister chromatid cohesion complex conveys clinical consequences distinct from Cornelia de Lange. *Journal of Medical Genetics*.

[25] Reichow B., Barton E. E., Boyd B. A., Hume K. (2012). Early intensive behavioral intervention (EIBI) for young children with autism spectrum disorders (ASD). *Cochrane Database of Systematic Reviews*.

[26] Peters-Scheffer N., Didden R., Korzilius H., Sturmey P. (2011). A meta-analytic study on the effectiveness of comprehensive ABA based early intervention programs for children with autism spectrum disorders. *Research in Autism Spectrum Disorders*.

[27] Martin C. L., Ledbetter D. H. (2017). Chromosomal microarray testing for children with unexplained neurodevelopmental disorders. *JAMA*.

[28] Gardner R. J. M., Amor D. J. (2018). Gardner and Sutherland’s Chromosome Abnormalities and Genetic Counseling. The Origins and Consequences of Chromosome Pathology. *Oxford Monographs on Medical Genetics*.

[29] Malan V., Vekemans M., Turleau C. (2006). Chimera and other fertilization errors. *Clinical Genetics*.

[30] Gug C., Stoicanescu D., Mozos I. (2020). De novo 8p21.3⟶ p23.3 duplication with t(4;8)(q35;p21.3) translocation associated with mental retardation, autism spectrum disorder, and congenital heart defects: case report with literature review. *Front Pediatr*.

[31] Gug C., Mozos I., Ratiu A. (2022). Genetic counseling and management: the first study to report NIPT findings in a Romanian population. *Medicina (Kaunas)*.

